# An Exon-Based Comparative Variant Analysis Pipeline to Study the Scale and Role of Frameshift and Nonsense Mutation in the Human-Chimpanzee Divergence

**DOI:** 10.1155/2009/406421

**Published:** 2009-10-22

**Authors:** GongXin Yu

**Affiliations:** Department of Biological Science and Department of Computer Science, Boise State University, Boise, ID 83725, USA

## Abstract

Chimpanzees and humans are closely related but differ in many deadly human diseases and other characteristics in physiology, anatomy, and pathology. In spite of decades of extensive research, crucial questions about the molecular mechanisms behind the differences are yet to be understood. Here I report *ExonVar*, a novel computational pipeline for *Exon*-based human-chimpanzee comparative *Var*iant analysis. The objective is to comparatively analyze mutations specifically those that caused the frameshift and nonsense mutations and to assess their scale and potential impacts on human-chimpanzee divergence. Genomewide analysis of human and chimpanzee exons with *ExonVar* identified a number of species-specific, exon-disrupting mutations in chimpanzees but much fewer in humans. Many were found on genes involved in important biological processes such as T cell lineage development, the pathogenesis of inflammatory diseases, and antigen induced cell death. A “less-is-more” model was previously established to illustrate the role of the gene inactivation and disruptions during human evolution. Here this analysis suggested a different model where the chimpanzee-specific exon-disrupting mutations may act as additional evolutionary force that drove the human-chimpanzee divergence. Finally, the analysis revealed a number of sequencing errors in the chimpanzee and human genome sequences and further illustrated that they could be corrected without resequencing.

## 1. Introduction

Chimpanzees (*Pan troglodytes*) and humans (*Homosapiens*) are each other's closest living relatives and yet the two primates differ enormously in many characteristics in physiology, anatomy, and pathology [1]. Among the differences, those in the frequency and severity to deadly human diseases such as human immunodeficiency disease (HIV/AIDS), Alzheimer's disease, and *Plasmodium falciparum* malaria are especially intriguing. In the case of HIV/AIDS, the infections of HIV-1 rarely cause AIDS-like diseases in chimpanzees. Protective strategies appear to have evolved to pacify SIV/HIV [2]. Similarly, the great apes can be infected with viral hepatitis B and C but do not progress to chronic active hepatitis [3]. While the epithelial neoplasm such as carcinomas of the breast, ovary, lung, stomach, colon, pancreas, and prostate cause more than 20% of the deaths in modern human populations, the occurrence rates among the great apes are only ~2%–4% or lower [4, 5]. Chimpanzees also appear to be immune to *P. falciparum,* the most aggressive and acutely life-threatening malaria for humans [6–8]*. *Finally, the great apes show no sign of the complete pathological lesions of Alzheimer's disease [9].

 The benefit of the comparative analysis of the closely related species is obvious. Through such analyses, it is possible to detect key differences that propelled the chimpanzee-human differentiation. The analysis thus could led to an improved understanding of the molecular mechanisms behind these differences and eventually to new therapies [10]. For example, the HIV-infected chimpanzee differs from HIV-infected human individuals in the following several aspects: (1) a low level of T cell activation and bystander apoptosis in SIV/HIV-infected chimpanzees with no evidence of immunodeficiency [11]; (2) a rapid establishment of an anti-inflammatory environment, which may prevent the host from developing the aberrant chronic T cell hyperactivation, a hallmark of progression to AIDS during HIV-1 infection [12]; and (3) An absence of overt CD4+ T cell loss. The nonpathogenic characteristics hence highlight a protective role of down-regulated T cell activation and the establishment of anti-inflammatory profiles early on in immune responses. It is possible that such protection allows the natural host to accept the virus that hence can survive in a symbiotic state. Knowledge about the molecular mechanisms of such protection would allow the development of drugs as well as treatment plans that can imitate biological processes that occur in the HIV-infected chimpanzees. The consequence is to bring the disease under control [13]. The strategy could apply equally well to other differentially evolved human diseases, for example, viral hepatitis B and hepatitis C, should it prove successful. Chimpanzees display a similar response to viral hepatitis B and C as they do to the HIV/AIDS [14] where infected chimpanzees do not progress to the chronic active hepatitis [3].

 The availability of completely sequenced human genomes [15, 16] and that of chimpanzees [1] would have an immense impact on the comparative human-chimpanzee analyses. For the first time, nucleotide-by-nucleotide comparisons can be performed at whole genome levels. Already such analyses have resulted in some novel observations and in-depth understandings. First, the two closely related species differ significantly in chromosome structures. Cheng et al*.* (2005) discovered difference in the contents of segmental duplications [17]. About 33% of the human duplications, including some human disease-causing duplication, are not duplicated in the chimpanzees. Harris et al. (2007) reconstructed ancestral states and the structural evolution of the genomes and identified 130 human-specific breakpoints due to rearrangements at an intermediate scale (10 kilobases to 4 megabases) [18]. Recently, Kehrer-Sawatzki and Cooper (2008) revealed a strong spatial association between primate-specific breakpoints and segmental duplications (SDs) [19].

 Chimpanzees and humans were also found to differ in adaptive gene evolution. Clark et al. (2003) observed nonneutral evolution from human-chimpanzee-mouse orthologous gene trios where significantly different patterns of substitution were detected in the human lineage where accelerated evolution was founded in several functional classes, including olfaction and nuclear transport [10]. Marques-Bonet et al. found an association between chromosomal rearrangements and genic evolution in human and chimpanzee [20–22]. Genes located in the rearranged chromosomes that differentiate the genomes of humans and chimpanzees presented lower divergence than genes elsewhere in the genome. These observations were further supported by the chimpanzee sequencing project and the subsequent human-chimpanzee comparative analysis [1]. The lineage-specific adaptive evolution was detected where affected genes differed in the magnitude, regional variation, and the strength of positive and negative selection. In spite of the extensive research, many questions remain, especially impacts of genetic mutations from recent human and chimpanzee evolution. Here I reported a novel computational platform named *ExonVar* for human-chimpanzee comparative analysis with a new perspective: comparatively analyzing mutations and their impacts on exon structures, specifically those that cause frameshift and nonsense mutations. The objective is to assess the scale and potential impacts of these mutations on human-chimpanzee divergence.

 The work was started because of the lack of appropriate computational procedures for such analysis although a few of comparative human-chimpanzee genome analysis methods have been developed. Hahn and Lee are the first group to develop a procedure that can be applied for a large scale analysis of first frameshift [23] and then nonsense mutations [24]. Their approach, relying on mRNA-genome sequence alignments, has its limitation mainly because of incomplete genome coverage of existing mRNA data especially from chimpanzees. Puente et al. (2006) developed the similar procedure but with a specific focus on cancer genes [25]. Recently, Wetterbom et al. (2009) devised a procedure for a genomewide analysis of chimpanzee genes with premature termination codons [26]. In this approach, exons downloaded from Ensembl and then translated. Exons were subsequently concatenated and scanned for premature termination codons. A main concern for this approach is that the accuracies in the exon boundary prediction will likely have a negative impact on the analysis. Despite numerous developments of useful tools, predicting exons and their precise exon–intron boundaries are still a challenging task [27–29]. An incorrect prediction would introduce incorrect open reading frames, thus false positive results.

 It was motivated by our early success in the development of GenVar and by the observations that gene functional modification or inactivation plays a crucial role in human genome evolution. GenVar is a computational pipeline to comparatively analyze closely related bacterial genomes for variant-pathogenesis association studies [30]. The pipeline is unique in that it can analyze sequence variations such as those that cause frameshift, nonsense and indel mutations at the genome scale and within the context of closely related bacterial species. From the analyses, mutations derived from recent evolution can thus be identified, promising a better understanding of the molecular basis underlying differentially evolved phenotypes [31].

It was also motivated by the fact that genetic changes can result in the acquisition of novel phenotypic traits, manifested by small jaws and teeth, weakened jaw muscles, decreased smell sensitivity, and reduced body hair. This is what so called a *“less-is-more”* model [31, 32]. For example, the human-specific 2-bp deletion in the coding region of MYH16, a sarcomeric myosin gene, resulted in a frameshift and was linked to the reduction of jaw muscles that allowed humans to have bigger brains [33]. The single base-pair substitution introduced a premature TGA termination codon in the human type I hair keratin gene [34]. The resulting gene inactivation has been suggested to evolve smooth, hairless skin in humans for enhanced thermoregulation [35] and/or for reduction of parasite loads [36]. Recently, multiple human-specific nonsense mutations have been identified where abolished or modified functions were predicted in the affected genes [24].

 It is thus hypothesized that functional modification or inactivation likely assumes greater roles in human-chimpanzee differentiation. GenVar is a powerful tool to test the hypothesis since it can assess the genome variations at a global scale. GenVar, however, is a CPU-demanding pipeline that presents a significant challenge in the human-chimpanzee genome study. First, there was an impractically high computational cost and running time due to the complexities of the human, chimpanzee and other eukaryotic genomes. The genomes are massive; human genome, for example, consists of over three billion base pairs (bps) with over 30,000 genes and 61,318 transcripts whereas average bacterial genomes are about 3 million bps with around 3,000 genes/proteins. The genes from these genomes are further more complicated than their bacterial counterparts. They can reach up to millions of base pairs but only tiny portions are actual coding sequences that are further imbedded in vast arrays of non-coding DNA. Many genes are alternatively spliced, forming multiple protein products [37, 38]; moreover, duplication is a ubiquitous phenomenon in humans and many other eukaryotic genomes and can reach a level with that bacterial genomes can barely match [39, 40].

 The complexities consequently result in genomic DNA inputs with gigantic sizes and database inputs with heterogeneous protein components, thus slowing the GeneWise-based analysis process and complicating result interpretations. This can be manifested by our earlier experiments. A small-sized human chromosome (e.g., chromosome 21) needs over three months of computational time in a Linux node with a dual Intel processor. By contrast, bacteria, with the same number of genes, need less than 24 hours [30]. Second, the result from the analysis was unreliable and inaccurate when GeneWise was applied to align the interrupted, mega-sized gene sequences (intron-exon structures) with their homologous proteins. The unreliability was obvious: approximately 24.48% of disrupted genes were revealed in human chromosomes 21 when compared to homologous proteins from humans, chimpanzees, and macaques but a majority of the disruption mutations turned out to be false positive when the alignments were manually examined.

*ExonVar* followed the same strategy as GenVar with critical modifications to overcome the challenge. First, all the analyses were performed on coding exons instead of genes. The coding exons are much smaller, even smaller than bacterial genes. With the divide and conquer strategy, an immediate result is an improved performance. With the same human chromosomes 21, the analysis can be finished within 24 hours at the same Linux node. The modifications, furthermore, come with an increased accuracy and simplified interpretation. All exon-disrupting mutations detected were found in their corresponding trace sequences. Finally, the human-chimpanzee analysis could be extended to include the genomes of macaques and other mammals to define lineage-specific sequence variants. With the computational pipeline, human and chimpanzee exons were analyzed. This paper presents a sample of total results to illustrate the analysis procedure and to assess the scale and biological impacts of the species-specific, exon-disrupting mutations.

## 2. Materials and Methods

### 2.1. Genome Data

Genome sequences of eight mammalian species were downloaded from Ensembl database in (2007) (ftp://ftp.ensembl.org/pub/). The downloaded data are release-46 (Table 1). Each genome is comprised of proteins, assembled chromosomes, and multiple MySQL files in the database. All genomes have completely assembled chromosomes with some variations at the time when the data were downloaded. For example, sequence data for humans consist of 22 autosomal chromosomes, 1 mitochondrion, 2 sex chromosomes, and three other unassembled DNA fragments. The MySQL files include several species-specific database tables including gene.txt.table, gene_stable_id.txt.table, exon.txt.table, exon_transcript.txt.table, transcript.txt.table, translation_stable_id.txt.table, and translation.txt.table. The purpose is to extract genomic information at the levels of genes, exons, and proteins, and to define the exon-gene and exon-protein relationships.

### 2.2. The ExonVar Implementation

*ExonVar* consists of three steps: first detect sequence mutations specifically those that cause frameshift and nonsense in the coding sequences are identified (i). Once identified, the occurrence patterns of the mutations were defined among the genomes of humans, chimpanzees, macaque and five other mammals (ii). Those that are specific to humans or chimpanzees were subsequently validated (In Silico) to tag those that are caused by sequencing errors (iii). Following sections give a detailed description about the procedures.

**Figure 1:**
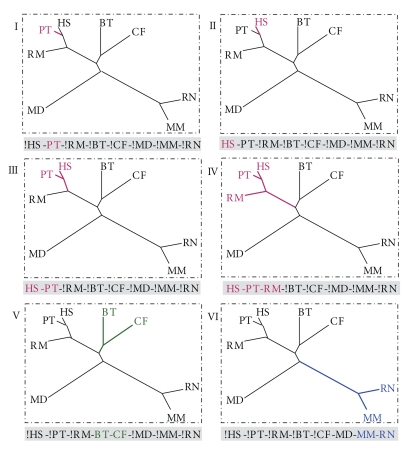
The species tree-based analysis procedure is to define occurrence patterns of the exon-disrupting mutations. The tree is drawn roughly based on a phylogenetic analysis of data from eight completely sequenced nuclear genomes [41]. Abbreviations of eight mammalian species are listed as followings: HS = *Homo sapiens*, PT = *Pan troglodytes*, RM = *Rhesus macaques*, BT = *Bos taurus*, CF = *Canis familiaris*, MD = *Monodelphis domestica*, MM = *Mus musculus*, and RN = *Rattus norvegicus*. Colored lines represent different lineages: the red for human-chimpanzee-macaque lineage; the green for dogs and cows; the blue for mouse-rat.

 (i) *Develop the exon-based, variant discovering procedure*. The development followed the analysis procedure described in GenVar but with a critical modification [30]. Instead of comparatively analyzing entire genes, which are hard to handle because of massive size and complicated structures, this procedure focuses solely on exons, the simplest and smallest coding units in eukaryotic genomes. Briefly, two required inputs are first established; one is *Exon-based* genomic DNA input and another is the *Exon-based Peptide DataBase* input (*ExPepDB*). The genomic DNA input was defined as an extended area of the predicted exons where 100 base pairs were added at both 3′ and 5′ ends. The *ExPepDB*, established for each genomic DNA input, consists of homologous peptide exons from all included genomes. The peptide exons are the amino acid sequences of the exons, translated by an in-house developed, Perl-based program. The program basically performed BLAST analyses against species-specific protein databases and subsequent protein-exon sequence mapping. Note that data downloaded from Ensembl have explicit specifications about protein-exon relationships. The program takes the relationship as a constraint. From that, amino acid sequences of any possible overlapped coding exons can be unequivocally determined. Once the two required inputs are established, they are comparatively analyzed using GeneWise [42–44] to identify sequence variations.

 (ii) *Identify lineage-specific exon-disrupting mutations*. To identify human- or chimpanzee-specific mutations, a tree-based evolutionary scheme was devised based on a previously published mammalian species tree [41]. In this scheme, macaques, cows, dogs, opossums, mice and rats, which are closely related, but phylogenetically outside of the chimpanzee and human clade, are used as out-groups (Figure 1). Mutations that follow the species tree were considered to be lineage-specific (panels I to V). Among them, those that are specific to chimpanzees or humans (in panel I and II) were defined as chimpanzee- or human-specific respectively.

 (iii) *Insilico validate the lineage-specific exon-disrupting mutations*. The first step is to establish species-specific trace databases for humans and chimpanzees. The trace data were downloaded from NCBI Trace archive (ftp://ftp.ncbi.nih.gov/pub/TraceDB/). The human-specific trace database covered 188, 150, 586, 226 base pairs (bp) of trace sequences from the diploid genome sequences of Dr. Craig J. Venter, Dr. James D. Watson, and other human sequencing projects, and has a total coverage of 58X based on the human genome size of 3, 253, 037, 807 bps. The chimpanzee-trace database is much smaller, which includes 43, 176, 085, 998 bp trace sequences from *Pan troglodytes*, a coverage of merely 15X. The database was extended to include 321, 835, 223 bp trace sequences from pongo_pygmaeus (sequencing project) and 15, 420, 096, 318 bp trace sequences from pongo_pygmaeus_abelii (sequencing project). The extension led to a 5.4X increase in the genome coverage (see results and discussion for reasoning to include Pongo sequences).

 The second step is to select query sequences. The objective is to validate the frame-disrupting mutations in target exons. The query sequences are the orthologous exons from the closest related genome, for example, exons in humans to those in chimpanzees or vice verse. The queries are then used to search the species-specific trace DNA database. Trace sequences with at least a 95% sequence identity are extracted. They were named candidate trace sequences to represent those that were sequenced directly from the chromosomal regions where the disrupted exons are detected. The candidate trace sequences, along with query-specific *ExPepDB*, are comparatively analyzed with GeneWise. The GeneWise alignments are evaluated with a set of predetermined rules. The assumption underlying the rules is that mutations derived from evolution (accepted mutations) will lead to the consistent occurrence of the variations among the candidate trace sequences whereas sequencing errors to randomness if ever occurred. Specifically, the rules say that for a given query,

- there will be at least *n* candidate trace sequences that have a coverage on the regions where the exon-disrupting mutations occur where *n* ≥ 2 and
- among the *n* candidate trace sequences at least *m* display exactly identical mutations in types and positions where *m* ≥ 2;
- none of the candidate trace sequences show a complete alignment with their orthologous sequences where no disruptions were displayed.

If all statements described above are evaluated as true, the status of the mutations will be considered as true and recognized as accepted mutations. Otherwise, the status of the mutations will be recognized as either false (statement I is true, but both II and III are false) or undetermined (statement I is false or both statement I and II are true but III is false).

### 2.3. Program Implementation

All implementations were based on the PERL script language. Wise2 was downloaded from EMBL-EBI (http://www.sanger.ac.uk/Software/Wise2/). *ExonVar* is available to noncommercial users upon request.

## 3. Results and Discussion

### 3.1. Comparative Analysis of the Exon-Disrupting Mutations

Following the procedure of *ExonVar*, all 504,862 predicted human and chimpanzee exons were analyzed. The analysis revealed significant differences between chimpanzees and humans. The first is the number of the disrupted exons: there are 1,931 disrupted exons in humans while that number is 3,742, approximately doubled in chimpanzees. The second is the occurrence patterns of the exon-disrupting mutations, which describes how the mutations are distributed among the genomes. In the human genome, only a small portion of the mutations are involved in cross-species variation between humans and chimpanzees. In chromosome X, for example, the number is 7.3% (eight out of 109 of the disrupted exons). Examples include HUMAN_1_851437 (solute carrier family 26, member 9 isoform A), HUMAN_1_837766 (calcium activated chloride channel 3 precursor) (Table 2). The exon-disrupting mutations are human-specific where they were detected in humans only. A majority of the disrupting mutations are, however, not species-specific. For instance, the exon-disrupting mutations in HUMAN_X_817906 (cancer/testis antigen 2) were detected by another human exon; that in HUMAN_X_806072 (intestinal protein OCI-5) by an exon from the dog genome, and that in HUMAN_X_815259 (green-sensitive opsin) by an exon from the macaque genome.

 On the contrary, a majority of the exon-disrupting mutations in chimpanzees were involved in human homologues, for example, 59.7% (114 out of 193 disrupted exons) in chromosome X. Among the exons, CHIMP_X_104156 (ADP-ribosylation factor-like protein 13A), CHIMP_X_99308 (Cylicin-1), CHIMP_X_146199 (AF4/FMR2 family member 2), CHIMP_X_144941 and CHIMP_X_144913 (melanoma-associated antigen C3) have disrupted reading frames when compared to their human and macaque orthologs, thus specific to chimpanzees. A similar phenomenon was found in other chromosomes (data not shown). Note that the exon names were designed for clarity and consistency where exon identification numbers from Ensembl database, for example, 142174 in CHIMP_X_142174, were used as bases with the prefixes of species abbreviations (CHIMP) and chromosome numbers (X).

### 3.2. Validation of the Species-Specific Exon-Disrupting Mutations

The exon-disrupting mutations can come from either sequence artifacts or accepted mutations [30]. With chimpanzee-specific trace databases, a total of 189 chimpanzee-specific frame-disrupted exons were examined with the predetermined rules. Case studies are represented here to illustrate the rules and their applications.

**Case 1**

**Figure 2:**
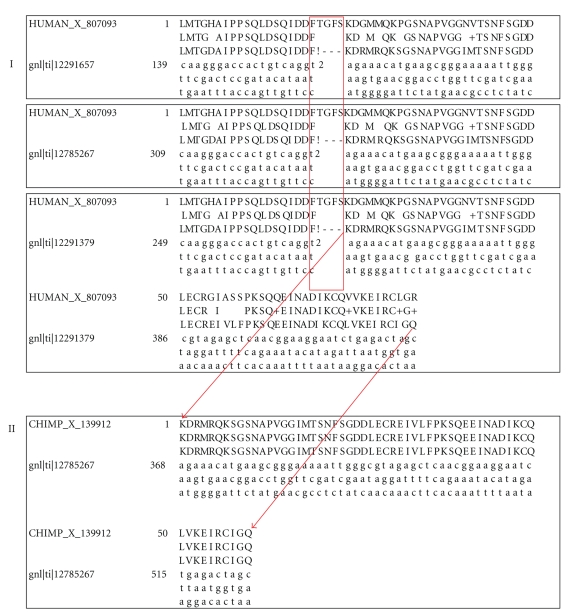
Validation of the chimpanzee-specific frameshift mutation in CHIMP_X_139912 (cancer/testis antigen CT45): (I) sequence alignments between the human orthologous exon and three candidate seed sequences from chimpanzee-specific trace database (gn1 |ti| 1278267, gn1 |ti| 12291279, gn1 |ti| 12291657). (II) sequence alignment between the chimpanzee exon and one of its candidate trace sequences. “!” represents frameshift mutations.

One chimpanzee-specific frameshift mutation was detected in CHIMP_X_139912 (cancer/testis antigen CT45). A total of 62 candidate trace sequences were extracted from the chimpanzee-specific trace database. An identical frameshift was revealed in all 62 sequences (Figure 2). Rules applied here are the following: (1) multiple candidate trace sequences were identified; (2) the exact same mutations in at least two seed sequences were observed; (3) no candidate trace sequences showed complete (undisrupted) open reading frame. The conclusion is that the frameshift mutation can be accepted as an adapted mutation derived from the recent human-chimpanzee evolution.

**Case 2**

**Figure 3:**
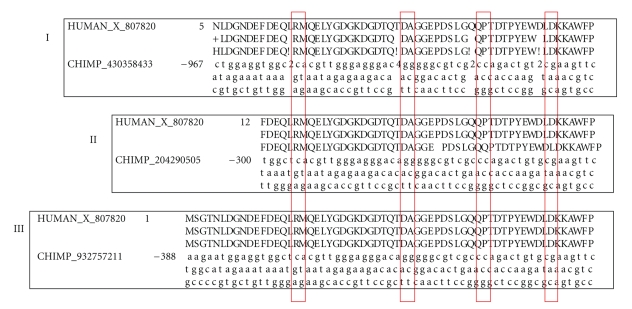
The validation of the chimpanzee-specific frameshift mutations in CHIMP_X_142174 (HIV Tat-specific factor (1): sequence alignments between the human orthologous exon and the candidate trace sequences from chimpanzee (I) and (II) and that from *pongo pygmaeus abelii * (III).

Four chimpanzee-specific frameshift mutations were detected in CHIMP_X_142174 (HIV Tat-specific factor (1) eight trace sequences were identified from the chimpanzee-specific trace database and only one was revealed to have the frameshift mutations. Some of the sequences had perfect alignments with its human and macaque orthologs (Figure 3). Rules applied here are the following: (1) Identified multiple candidate trace sequences; (2) observed the frameshift mutation in one candidate trace sequence only, which I believed to be the seed sequence that was used for the original genome assembly; and (3) detected undisrupted open reading frames from multiple candidate trace sequences including one from *Pongo pygmaeus abelii*. The conclusion is that the frameshift mutations were rejected and the disrupted exon was predicted to be due to sequencing artifacts.

Exon-disrupting mutations were also validated in the genes or homologues of cancer/testis antigen CT45-3, melanoma-associated antigen C3, the inhibitor of growth family, member 1, protocadherin-11 X-linked precursor, potassium channel tetramerisation domain containing 9-like, testis-expressed sequence 13A protein, tumor necrosis factor receptor superfamily member 18 precursor, heat-shock protein beta-7, eyes absent homolog 3, (EC 3.1.3.48), neuroblastoma breakpoint family, member 11, tropomyosin alpha-3 chain (Tropomyosin-3), voltage-dependent R-type calcium channel subunit alpha-1E, ADP-ribosylation factor-like protein 13A, SOX-13 protein (Type 1 diabetes autoantigen ICA12) (Islet cell antigen 12) (Table 3) and 36 other chimpanzee genes (data not shown).

Including trace sequences from two *Pongo* sequencing projects increased the genome coverage and enhanced the capability in the validation of the exon-disrupting mutations in the chimpanzee genome. For example, two frameshift mutations were found in CHIMP_1_235104, an exon of SOX-13 protein gene but only one chimpanzee candidate trace sequence was extracted from the chimpanzee-specific trace database. Fortunately two other candidate trace sequences were identified from the *Pongo pygmaeus abelii* trace database. All three candidates bear the same frameshift mutation, leading us to consider the frameshift as an accepted mutation. In CHIMP_1_178077, an exon for heat-shock protein beta-7, one homologous trace sequence from *Pongo pygmaeus abelii* was identified and found to share a nonsense mutation with three other chimpanzee trace sequences. A similar phenomenon was observed in many other exon-disrupting mutations in chimpanzee exons such as CHIMP_1_229134, an exon for tropomyosin alpha-3 chain (Tropomyosin-3), and CHIMP_X_91363, an exon for inhibitor of growth protein 1 homolog. On the other hand, differences were also observed between chimpanzees and pongos*.* For instance, a frameshift mutation was detected in 14 chimpanzee candidate trace sequences but not in any of the five pongo candidates. The observation is not surprising considering an approximate 13.04 Mya to 17.74 Mya differentiation time between *Pan troglodytes* and* Pongo pygmaeus,* a more distant relationship than between *Pan troglodytes* and* Homo sapiens* (a range of 5.65 Mya to 8.27 Mya) (http://www.timetree.org/) [45].

In summary, the analysis found an apparent asymmetry where more species-specific, exon-disrupting mutations were detected in chimpanzees than those in humans. One of the possible assumptions is that there is a bias towards detecting exons that are intact in humans because of more careful/complete annotations. Indeed, errors in annotations were detected in CHIMP_X_142174 and many other chimpanzee exons. We expected, however, such annotation errors would have limited impacts on the asymmetry because of our experimental design where all exons were extended by 100 base pairs at both end of the exons and then comparatively analyzed. The results were further Insilico validated.

### 3.3. The Structural Impacts

**Figure 4:**
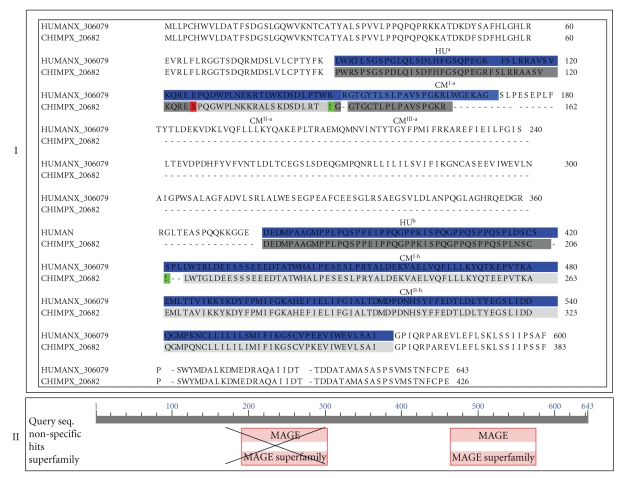
A comparative display of the melanoma-associated gene C3 in humans and chimpanzees: exon-disrupting mutations (I) and resulting domain structures (II). Note: exons were named in the format of A^B^ where A is the short abbreviation of mammalian species and B is the index of the exons. For example, HU^a^ is the first target exon of the human melanoma-associated gene C3 and highlighted by blue, CM^a-I^, CM^a-II^, CM^a-III^ are the three exons derived from HU^a^ due to the mutations. The neighboring chimpanzee exons were highlighted by dark and light gray alternatively. The subscript “X” represents nonsense mutation and “!” represents frameshift mutations (a). The cross represents the truncated part of the protein sequence and MAGE domain (b).

The lineage-specific exon-disrupting mutations were detected on many coding exons from the chimpanzee genome. Several examples were presented here to illustrate their potential structural and biological impacts, first through structure analysis of the affected genes and their resulting proteins, and then via the analyses of biological functions of the genes. Indeed, a combining effect of nonsense, frameshift, and exon missing mutations (data not shown) had completely reshaped the structure of the chimpanzee melanoma-associated gene C3 (Figure 4). Its exons were fractured where two human exons were split into a total of five chimpanzee ones (Figure 4(I)). Furthermore, the resulting protein sequence lost one of two functional MAGE domains (Figure 4(II)). Similar impacts were observed on melanoma-associated gene C2, SOX-13 protein (Type 1 diabetes autoantigen ICA12), tumor necrosis factor receptor superfamily member 18 precursor and many other genes where the overall structures of genes and their protein products were affected.

### 3.4. Functional Impacts

Genes with the species-specific exon-disrupting mutations appear to be involved in some critical physiological and biological processes. In chimpanzees, for example, affected genes included those encoding a series of the melanoma-associated antigens, for example, MAGE-C1 antigen, MAGE-3, MAGE-C2 and MAGE-1 antigen in addition to MAGE-C3 described above. Additional affected genes include those for the inhibitor of the growth family, member 1 (ING1), the testis-expressed sequence 13A protein, neuroblastoma breakpoint family, member 11, the tumor necrosis factor receptor superfamily member 18 precursor (TNFSF18), SOX-13 protein, heat-shock protein beta-7 (HSPB7), Eya3 and protocadherin-11 X-linked precursor (PCDH11X). These are genes/homologues with crucial roles in cell differentiations and developments.

 The melanoma-associated genes are unique in that they are strictly tumor- and testis-specific and furthermore, their protein products can be recognized by autologous cytolytic T lymphocytes in many human tumors [47]. The inhibitor of the growth family, member 1 (ING1) is a breast cancer suppressor gene [48]. The acute expression of transfected constructs encoding ING1 inhibited cell growth while chronic expression of ING1 antisense constructs promoted cell transformation [49]. The gene encoding the testis-expressed sequence 13A protein is a spermatogonically-expressed, germ-cell-specific genetic factor. Cytogenetic analysis indicated that the gene is located near breakpoints on the X chromosome in the azoospermic patients with X-autosome translocations [50], suggesting that it may be one of the direct genetic risk factors for azoospermia. Gene of the neuroblastoma breakpoint family, member 11 has been implicated in recent genome duplications, illegitimate recombination, and chromosomal translocation in a neuroblastoma patient [51].

 Tumor necrosis factor receptor superfamily member 18 precursor (TNFSF18) is a member of the tumor necrosis factor (TNF) and TNF receptor (TNFR) gene superfamilies. Data accumulated from experiments indicated that it plays important roles in regulating cell proliferation, differentiation, and survival. This was manifested from Gurney's experiment [52] where cotransfection of the tumor factor receptor and its ligand in Jurkat T leukemia cells inhibited antigen-receptor-induced cell death. Recently Kim et al. (2006) showed the role of the gene in mediating the inflammatory activation of macrophages that can destabilize atherosclerotic plaques [53]. Bae et al., (2008) showed that TNFSF18 may be involved in the pathogenesis of inflammatory diseases [54]. The stimulation of this gene induced the expression of pro-inflammatory cytokines and matrix metalloproteinase (MMP)-9 and up-regulated ICAM-1 expression levels.

 Gene of SOX-13 protein is a transcription factor of the sex-determining region [SRY]-type high mobility group [HMG] box) family. Among other regulatory functions, Sox13 is the first lineage specific gene identified that modulates T cell lineage development. This gene promotes gamm/adelta T cell development while opposes alpha/beta T cell differentiation [55]. Mice deficient in Sox13 expression exhibited impaired development of gamma/delta T cells but not alphabeta T cells. Previous research suggested a possible role of the heat-shock protein beta-7 (HSPB7) in cardiovascular development. Krief et al. (1999) found that it was selectively expressed in cardiovascular and insulin-sensitive tissues [56]. In obese Zucker rats, its mRNA was increased in skeletal muscle, brown, and white adipose tissues but remained unchanged in the heart. Finally, few data were available for functions of Eya3 except the fact that the gene is expressed in the developing eye including the branchial arches and CNS but not cranial placode [57].

**Figure 5:**
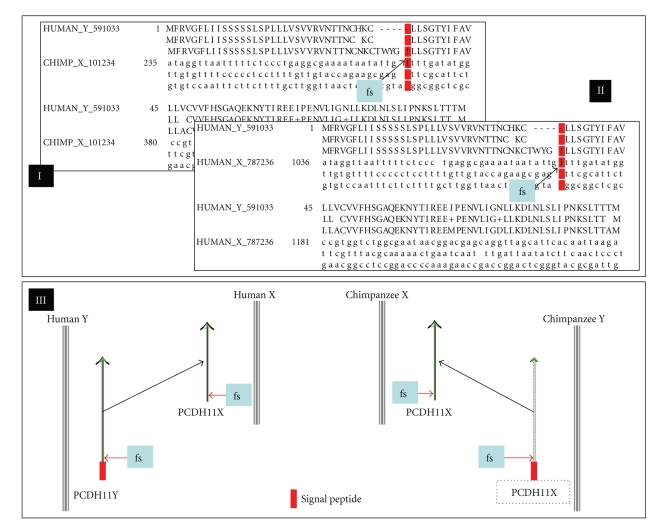
Impacts of exon-disrupting mutations on the structure of the X-linked protocadherin-11 gene in chimpanzees (I) and humans (II): A frameshift mutation results in the deletion of N-terminal 29-residue signal peptides in the X-linked protocadherin-11 proteins in chimpanzee and human genomes (III). The dotted box indicates the absence of Y-linked protocadherin-11 in chimpanzees. Signal peptide was predicted through SignalP 3.0 Server (http://www.cbs.dtu.dk/services/SignalP/) [46].

 Among these exon-disrupting mutations, those on protocadherin-11 X-linked precursor (PCDH11X) are especially inspiring. Single frameshift mutations were detected in PCDH11X of both chimpanzee and human genomes when compared to their human Y-linked paralogue (PCDH11Y). The mutations resulted in the loss of the N-terminal 29-residue signal peptide in the X-linked protocadherin-11 proteins (Figure 5), suggesting that PCDHY and PCDHX may function at different cellular locations and differentially regulated. Indeed, Blanco et al. (2000) observed differential regulation in a pluripotential cell line. PCDHX predominated before retinoic acid treatment whereas PCDHY predominated after retinoic acid treatment [58]. In addition, Yang et al. (2005) observed that the PCDHY is selectively expressed in apoptosis- and hormone-resistant human prostate cancer cells [59]. An upgraded expression of PCDHY activated WNT signaling and drove neuroendocrine transdifferentiation. The observations suggested a significant different role of the PCDHY for the process through which prostate cancers progress to hormone resistance in humans. No Y-linked protocadherin-11 was observed in the chimpanzee genome. It will be interesting to investigate the biological impacts of the lack of the Y-linked protocadherin gene, structural changes of the melanoma-associated antigens and many others on physiological properties, especially disease responses of humans and chimpanzees.

 So far, a total of 19 human-specific, exon-disrupting mutations were examined and among them, those in HUMAN_1_851772, HUMAN_1_837766 and HUMAN_1_851437 were validated (Table 3). HUMAN_1_851772 is an exon for the gene that encodes complement receptor type 1 precursor (C3b/C4b receptor) (CD35 antigen). The gene was identified as the immune-adherence receptor for the complement fragments C3b and C4b (CR1) [60]. Its deficiency in erythrocytes was associated with systemic lupus erythematosus [61]. HUMAN_1_851437 is an exon of gene for solute carrier family 26, member 9 isoform A (SLC26A9). The analysis of SLC26A9 expression patterns with homeostatic stress suggested a important role in the mediation of the response of the airway to stress [62]. HUMAN_1_837766 is an exon of gene encoding calcium activated chloride channel 3 (CLCA3). As early as 1999, CLCA3 was characterized as a truncated, secreted member of the human family of Ca^2+^-activated ^Cl-^channels [63]. Compared to its 125-kDa transmembrane paralogous protein, the CLCA3 encodes a 37-kDa glycoprotein that corresponds to the N-terminal extracellular domain, suggesting that it does not act as a channel protein but has distinct, yet unidentified functions.

### 3.5. Summary

In spite of decades of extensive research, some questions remains about what is exactly behind the differences between two closely-related primates [1]. The availability of completely sequenced genomes of humans and chimpanzees allows nucleotide-by-nucleotide comparisons at whole genome scale. Yet, an immense technique challenge remains due to the complexities of human and chimpanzee genomes. With the divide and conquer strategy, *ExonVar* is expected to provide new perspective in the field. Instead of comparatively analyzing eukaryotic genes, all the analyses focused exclusively on exons, the simplest and smallest coding units in the genomes.

 With all the exons on human and chimpanzee genomes, this analysis demonstrated that the newly developed pipeline could improve performance and increase accuracy, and reveal significant differences between humans and chimpanzees. A large number of species-specific, exon-disrupting mutations were revealed in chimpanzees. The exon-disrupting mutation fractured exons, truncated protein domains, and thus forced the structural shifts of many chimpanzee genes away from that of humans. The differentially evolved exons are further involved in many crucial biological processes such as T cell lineage development, the pathogenesis of inflammatory diseases, antigen-receptor-induced cell death, cancer progression and many other important biological processes. It is thus hypothesized that the frameshift and nonsense mutations could play a great role in the human-chimpanzee divergence. Finally, the analysis discovered a number of sequencing errors but a majority of them can be corrected without resequencing. The pipeline thus will be valuable in improving genome annotations, enhancing understanding of human evolution, and eventually, providing drug candidates and strategies for better disease treatments.

## Figures and Tables

**Table 1:** Eight assembled/partially assembled mammalian genomes.

Genomes	Version	Species symbol	Commonname	Assembled chromosomes	Exon
*Homo sapiens*	NCBI36.46	HS	Human	1-22, X, Y, MT, c22-H2, c5-H2, c6-COX, c6-QBL	267971
*Pan troglodytes*	CHIMP2.1.46	PT	Chimpanzee	1, 2A, 2B, 3-22, X, Y, MT, nonchrom	236891
*Rhesus macaque*	MMUL_1.46	RW	Macaque	1-20, X, MT, nonchrom	242505
*Mus musculus*	NCBIM36.46	MM	Mouse	1-19, X, Y, MT, nonchrom,	229909
*Rattus norvegicus*	RGSC3.4.46	RN	Rat	1-20, X, MT, nonchrom	241761
*Canis familiaris*	BROADD2.46	CF	Dog	1-38, X, MT, nonchrom	211964
*Bos taurus*	Btau_3.1.46	BT	Cow	1-30, nonchrom	215534
*Monodelphis domestica*	BROADO5.46	MD	Opossum	1-8, X, MT, Un, nonchrom	244769

**Table 2:** The occurrence patterns of exon-disrupting mutations on chromosome X of humans and chimpanzees.

Frameshift	Premature stop codon	Subject peptide exon	Alignment score	Function annotation
**EXON_ID: HUMAN_X_792407 Testis-expressed sequence 13A protein**				

104351239	-	CHIMP_X_110562	715.92	Testis-expressed sequence 13A protein
-	-	HUMAN_X_792407	607.24	Testis-expressed sequence 13A protein
-	-	CHIMP_X_110628	435.54	Testis-expressed sequence 13A protein
-	-	MONK_X_10782	288.8	Testis-expressed sequence 13B protein
-	-	DOG_X_198644	272.73	∖N

**HUMAN_X_806072 Glypican-3 precursor (Intestinal protein OCI-5)**				

132662038	-	DOG_X_194924	136.67	Glypican-3 precursor
-	-	HUMAN_X_806072	117.76	Glypican-3 precursor
-	-	MONK_X_12252	117.76	Glypican-3 precursor
-	-	MOUSE_X_2142643	116.89	Glypican-3 precursor

** HUMAN_X_817906 Cancer/testis antigen 1B**				

153468119	-	HUMAN_X_818108	231.77	Cancer/testis antigen 2
-	-	HUMAN_X_817906	216.52	Cancer/testis antigen 1B
-	-	HUMAN_X_817908	117.27	Cancer/testis antigen 1B
-	-	MONK_X_13842	102.91	∖N
-	-	MONK_X_13845	84.38	∖N
-	-	HUMAN_X_818110	78.11	Cancer/testis antigen 2
-	-	CHIMP_X_160765	78.02	Cancer/testis antigen 2
-	-	CHIMP_X_160692	52.31	∖N

** HUMAN_X_815259 Green-sensitive opsin**				

-	153149508	MONK_X_13549	298.36	∖N
-	-	HUMAN_X_815212	223.34	Green-sensitive opsin
-	-	HUMAN_X_815259	223.34	Green-sensitive opsin
-	-	CHIMP_X_157000	204.73	Red-sensitive opsin
-	-	HUMAN_X_815154	204.73	Red-sensitive opsin
-	-	DOG_X_197028	201.64	Red-sensitive opsin
-	-	RAT_X_186855	200.53	opsin 1 (cone pigments)
-	-	OPOSSUM_X_8729	199.61	∖N

** HUMAN_X_781680 Translationally-controlled tumor protein (TCTP) (p23)**				

-	-	CHIMP_X_92139	367.27	TPT1-like protein
-	-	HUMAN_X_781680	367.27	TPT1-like protein
71297119 & 71297149	-	MONK_1_81482	343.44	Translationally-controlled tumor protein (TCTP) (p23)
71297119 & 71297149	-	MONK_9_138306	335.33	∖N
71297119 & 71297149	-	MOUSE_6_2214409	326.93	Tumor protein, translationally-controlled 1
71297119 & 71297149	-	MOUSE_9_2157034	323.69	∖N
71297119 & 71297149	-	RAT_1_18606	319.31	∖N
-	-	OPOSSUM_1_184539	318.51	∖N
-	-	OPOSSUM_4_115959	302.14	∖N
71297122 & 71297149	-	RAT_16_40873	298.6	∖N
71297119 & 71297149	-	RAT_7_212305	293.94	∖N

** HUMAN_X_805870 Heparan-sulfate 6-O-sulfotransferase 2 (EC 2.8.2.-) (HS6ST-2)**				

131920284	-	MONK_X_12231	316.46	Heparan-sulfate 6-O-sulfotransferase 2
131920284	-	COW_Un_11290	273.56	∖N
131920284	-	DOG_X_154771	268.61	∖N
131920284	-	MOUSE_X_2142311	125.07	Heparan sulfate 6-O-sulfotransferase 2
131920284	-	RAT_X_171447	125.07	∖N
-	-	HUMAN_X_805870	72.68	Heparan-sulfate 6-O-sulfotransferase 2
-	-	CHIMP_X_136642	68.81	Heparan-sulfate 6-O-sulfotransferase 2

** HUMAN_1_851437 solute carrier family 26, member 9 isoform A**				

204150951	-	CHIMP_1_235519	351.23	Solute carrier family 26, member 9 isoform A
204150951	-	MONK_1_76747	337.22	Solute carrier family 26, member 9 isoform A
-	-	HUMAN_1_851437	252.06	Solute carrier family 26, member 9 isoform A
-	-	HUMAN_1_851439	252.06	Solute carrier family 26, member 9 isoform A

** HUMAN_1_837766 calcium activated chloride channel 3 precursor**				

-	86881166	CHIMP_1_224547	183.12	Calcium activated chloride channel 3 precursor
-	-	HUMAN_1_837766	157.82	Calcium activated chloride channel 3 precursor
-	86881166	COW_3_170345	139.02	Calcium activated chloride channel 3 precursor
-	86881166	COW_3_170045	138.65	Calcium activated chloride channel 3 precursor
-	86881166	DOG_6_162589	128.97	Calcium activated chloride channel 3 precursor
-	86881166	MOUSE_3_2282090	123.94	Calcium activated chloride channel 3 precursor
-	86881166	RAT_2_220515	118.93	Calcium activated chloride channel 3 precursor
-	86881166	MONK_1_36555	103.72	Calcium activated chloride channel 3 precursor

** HUMAN_X_785548 Putative P2Y purinoceptor 10 (P2Y10) (P2Y-like receptor)**				

78227761	-	RAT_X_146932	618.82	∖N
78227761	-	MOUSE_X_2200474	617.25	RIKEN cDNA A630033H20 gene [
-	-	HUMAN_X_785548	579.93	∖N
-	-	CHIMP_X_98456	563.37	∖N
78227761	-	OPOSSUM_X_50171	459.83	∖N
78227761	-	MONK_X_9587	432.12	Putative P2Y purinoceptor 10 (P2Y10)
78227761	-	HUMAN_X_785541	431.7	Putative P2Y purinoceptor 10 (P2Y10)
78227761	-	DOG_X_165474	419.31	∖N
78227761	-	COW_Un_11583	417.2	purinergic receptor P2Y, G—protein coupled, 10

** CHIMP_X_29616 Putative ataxin-3-like protein (Machado-Joseph disease protein 1-like)**				

-	-	CHIMP_X_29616	432.13	Putative ataxin-3-like protein
-	13263802	HUMAN_X_705429	423.98	Putative ataxin-3-like protein
-	13263802	HUMAN_X_705430	394.12	Putative ataxin-3-like protein
-	13263802	MONK_X_3550	376.14	Putative ataxin-3-like protein

** CHIMP_X_144931 Melanoma-associated antigen C3 (MAGE-C3 antigen)**				

141392714	-	HUMAN_X_809214	555.35	Melanoma-associated antigen C3
141392714	-	HUMAN_X_809208	365.01	Melanoma-associated antigen C3
-	-	CHIMP_X_144931	125.45	Melanoma-associated antigen C3
-	-	COW_15_185185	50.28	∖N

** CHIMP_X_146199 AF4/FMR2 family member 2 (Fragile X mental retardation protein 2 homolog) **				

148435573	-	HUMAN_X_809667	867.67	AF4/FMR2 family member 2
148435573	-	MONK_X_12858	854.06	AF4/FMR2 family member 2
-	-	CHIMP_X_146586	844	AF4/FMR2 family member 2
-	-	CHIMP_X_146199	840.35	AF4/FMR2 family member 2
148435573	-	DOG_X_165267	735.92	AF4/FMR2 family member 2
148435573	-	MOUSE_X_2153947	701.72	AF4/FMR2 family, member 2
148435573	-	RAT_X_179540	701.09	∖N
148435573	-	COW_X_46124	690.64	AF4/FMR2 family member 2
148435570	-	DANIO_14_239638	274.31	AF4/FMR2 family member 2

** CHIMP_X_99308 Cylicin-1 (Cylicin I) (Multiple-band polypeptide I) **				

83230590	-	HUMAN_X_786289	1347.4	Cylicin-1 (Cylicin I)
83230590	-	MONK_X_9728	1143.6	Cylicin-1 (Cylicin I)
-	-	CHIMP_X_99308	1123.9	Pan troglodytes cylicin, basic protein of sperm head cytoskeleton 1 (CYLC1), mRNA
-	-	MONK_X_9731	827.18	Cylicin-1 (Cylicin I)
-	-	MONK_X_9741	375.4	Cylicin-1 (Cylicin I)
-	-	MONK_X_9742	316.01	Cylicin-1 (Cylicin I)
83230593	-	DOG_X_87481	305.14	Cylicin-1 (Cylicin I)
-	-	DOG_X_87432	214.85	Cylicin-1 (Cylicin I)
-	-	RAT_X_149412	164.28	∖N

** CHIMP_X_142174 HIV Tat-specific factor 1 (Tat-SF1) **				

135897343	-	MONK_X_12551	108.9	HIV Tat-specific factor 1 (Tat-SF1)
135897343 & 135897388 & 135897418	-	HUMAN_X_807829	106.55	HIV Tat-specific factor 1 (Tat-SF1)
135897343	-	DOG_X_183603	94.43	
135897343	-	COW_X_29917	91.31	Hypothetical protein (Fragment)
135897343	-	RAT_X_174621	85.76	HIV TAT specific factor 1
135897343	-	COW_12_95371	82.45	∖N
135897343	-	MOUSE_X_2148052	77.47	HIV TAT specific factor 1
-	-	CHIMP_X_142174	61.13	HIV Tat-specific factor 1 (Tat-SF1)

Note: Numbers under the columns of frameshifts and premature stop codons are the chromosomal positions of the sequence mutations. “∖N” presents no annotation available from Ensemble database.

**Table 3:** Insilico validation of exon-disrupting mutations.

FR	PM	Subject	Score	Function description
**CHIMP_X_139912: PREDICTED: Pan troglodytes similar to Cancer/testis antigen CT45-3**				

135199011	-	MONK_X_12447	197.76	∖N
-	-	CHIMP_X_139912	159.41	Pan troglodytes similar to Cancer/testis antigen CT45-3
135199011	-	HUMAN_X_807093	140.8	Cancer/testis antigen CT45-4 (CT45-4)
135199011	-	HUMAN_X_806975	138.76	Cancer/testis antigen CT45
135199011	-	HUMAN_X_807082	138.76	Cancer/testis antigen CT45-4 (CT45-4)
135199011	-	HUMAN_X_807113	138.76	Cancer/testis antigen CT45-6 (CT45-6)
135199011	-	HUMAN_X_806962	137.58	Cancer/testis antigen CT45-1 (CT45-1)
135199011	-	HUMAN_X_806970	137.58	Cancer/testis antigen CT45
135199011	-	MONK_X_12441	136.48	∖N
135199011	-	CHIMP_X_140002	132.22	∖N

**CHIMP_X_144913: Melanoma-associated antigen C3**				

141390311	141390248	MONK_X_12774	153.22	∖N
141390314	141390248	HUMAN_X_809204	118.25	Melanoma-associated antigen C3
-	-	CHIMP_X_144913	104.42	Melanoma-associated antigen C3

**CHIMP_X_144941: Melanoma-associated antigen C3 **				

141392714 141393341	-	HUMAN_X_809214	811.08	Melanoma-associated antigen C3
141392714	-	HUMAN_X_809208	504.96	Melanoma-associated antigen C3
141392720	-	MONK_X_12788	498.06	Melanoma-associated antigen C2
141392720	-	HUMAN_X_809255	489.78	Melanoma-associated antigen C2
-	-	CHIMP_X_144941	415.82	Melanoma-associated antigen C3

**Chimp_x_91363: Homolog of Inhibitor of growth protein 1 **				

70808615 70808558	70808522 70808408	MONK_17_129198	197.85	Inhibitor of growth protein 1
70808615 70808558	70808522 70808408	MONK_17_129202	197.85	Inhibitor of growth protein 1
70808615 70808558	70808522 70808408	MONK_17_129209	197.85	Inhibitor of growth protein 1
70808615 70808558	70808522 70808408	CHIMP_13_65452	196.75	Inhibitor of growth protein 1
70808615 70808558	70808522 70808408	HUMAN_13_720109	196.75	Inhibitor of growth protein 1
70808615 70808558	70808522 70808408	HUMAN_13_720111	196.75	Inhibitor of growth protein 1
70808615 70808558	70808522 70808408	HUMAN_13_720113	196.75	Inhibitor of growth protein 1
70808615 70808558	70808522 70808408	HUMAN_13_720115	196.75	Inhibitor of growth protein 1
70808548	70808521 70808407	RAT_16_143085	188.7	Inhibitor of growth family, member 1
70808548	70808521 70808407	DOG_22_9324	186.9	∖N
70808548	70808521 70808407	MOUSE_8_2111806	185.9	Inhibitor of growth family, member 1
70808548	70808521 70808407	OPOSSUM_7_30919	185.56	Inhibitor of growth protein 1
70808548	70808521 70808407	COW_12_131559	181.68	Inhibitor of growth protein 1
-	-	CHIMP_X_91363	133.05	∖N
-	70808403	DANIO_9_335418	127.32	Hypothetical protein
-	70808403	DANIO_9_335470	127.32	Hypothetical protein
-	70808403	CHIMP_4_171949	112.69	Inhibitor of growth protein 2
-	70808403	COW_27_26326	112.69	∖N
-	70808403	DOG_16_29019	112.69	∖N
-	70808403	HUMAN_4_708541	112.69	Inhibitor of growth protein 2
-	70808403	MONK_5_172003	112.69	Inhibitor of growth family, member 1-like
-	70808403	OPOSSUM_5_33934	112.69	Inhibitor of growth protein 2
-	70808403	MOUSE_8_2166090	111.86	Inhibitor of growth family, member 2
-	70808403	RAT_16_84084	110.51	∖N
-	70808403	DANIO_1_359897	109.25	Hypothetical protein

**CHIMP_X_101234: Protocadherin-11 X-linked precursor **				

-	-	CHIMP_X_101234	516.68	Protocadherin-11 X-linked precursor
91237579	-	HUMAN_Y_591033	515.03	Protocadherin-11 Y-linked precursor
-	-	HUMAN_X_787233	509.39	Protocadherin-11 X-linked precursor
-	-	CHIMP_X_101543	479.57	Protocadherin-11 X-linked precursor
-	-	CHIMP_X_101362	473.55	Protocadherin-11 X-linked precursor

**CHIMP_X_110683: Potassium channel tetramerisation domain containing 9-like**				

104937007	104936803 104936929	HUMAN_9_756669	340.71	∖N
104937007	104936803 104936929	CHIMP_9_89777	334.23	∖N
104937147	104936946 104937072	HUMAN_8_631149	238.77	∖N
-	104936946	HUMAN_X_792440	182.69	Potassium channel tetramerisation domain containing 9-like
-	104936946	HUMAN_X_792443	180.93	Potassium channel tetramerisation domain containing 9-like
-	-	CHIMP_X_110683	93.45	Potassium channel tetramerisation domain containing 9-like

**CHIMP_X_110628: Testis-expressed sequence 13A protein **				

104742590	-	HUMAN_X_792407	564.62	Testis-expressed sequence 13A protein
-	-	CHIMP_X_110628	453.53	Testis-expressed sequence 13A protein

**CHIMP_1_136911 Tumor necrosis factor receptor superfamily member 18 precursor **				

1124826	-	HUMAN_1_800938	181.65	Tumor necrosis factor receptor superfamily member 18 precursor
-	-	CHIMP_1_136911	140	Tumor necrosis factor receptor superfamily member 18 precursor
1124826	-	MONK_1_14977	134.83	Tumor necrosis factor receptor superfamily member 18 precursor
-	-	MONK_1_14977	23.57	Tumor necrosis factor receptor superfamily member 18 precursor
-	-	CHIMP_1_136911	21.23	Tumor necrosis factor receptor superfamily member 18 precursor

**CHIMP_1_178077 Heat-shock protein beta-7 (hspb7) **				

-	16234672	HUMAN_1_816034	160.14	Heat-shock protein beta-7 (hspb7) (Cardiovascular heat shock protein)
-	16234672	HUMAN_1_816039	160.14	Heat-shock protein beta-7 (hspb7) (Cardiovascular heat shock protein)
-	-	CHIMP_1_178077	65.07	Heat-shock protein beta-7 (hspb7) (Cardiovascular heat shock protein)

**CHIMP_1_203439 Eyes absent homolog 3 (EC 3.1.3.48) **				

28236481	-	MONK_1_19843	67.86	Eyes absent homolog 3 (EC 3.1.3.48)
28236481	-	HUMAN_1_870638	67.24	Eyes absent homolog 3 (EC 3.1.3.48)
-	-	CHIMP_1_203439	58.79	Eyes absent homolog 3 (EC 3.1.3.48)

**CHIMP_1_226597 neuroblastoma breakpoint family, member 11 (NBPF11) **				

114514130	-	HUMAN_1_841875	116.78	Neuroblastoma breakpoint family, member 11 (NBPF11)
114514130	-	HUMAN_1_842031	116.78	Neuroblastoma breakpoint family, member 11 (NBPF11)
-	-	CHIMP_1_226597	96.53	Neuroblastoma breakpoint family, member 11 (NBPF11)
-	-	CHIMP_1_226350	92.54	Neuroblastoma breakpoint family, member 11 (NBPF11)

**CHIMP_1_229134 Tropomyosin alpha-3 chain (Tropomyosin-3) **				

133314785	-	HUMAN_1_843860	241.51	Tropomyosin alpha-3 chain
133314785	-	MONK_1_57153	224.17	Tropomyosin alpha-3 chain
-	-	CHIMP_1_229134	94.16	Tropomyosin alpha-3 chain

**CHIMP_1_233220 Voltage-dependent R-type calcium channel subunit alpha-1E **				
**(Voltage- gated calcium channel subunit alpha Cav2.3) **				
**(Calcium channel, L type, alpha-1 polypeptide, isoform 6) (Brain calcium channel II) **				

161366683	-	HUMAN_1_848676	364.5	Voltage-dependent R-type calcium channel subunit alpha-1E
161366683	-	MONK_1_84331	335.27	Voltage-dependent R-type calcium channel subunit alpha-1E
161366683	-	COW_16_164990	306.65	Calcium channel subunit alpha 1E
161366683	-	RAT_13_76001	300.14	Voltage-dependent R-type calcium channel subunit alpha-1E
161366683	-	MOUSE_1_2286080	299.66	Calcium channel, Voltage-dependent, R type, alpha 1E subunit
161366683	-	DOG_7_48804	298.94	∖N
-	-	CHIMP_1_233220	149.73	PREDICTED: Pan troglodytes similar to Voltage-dependent calcium channel alpha-1E-3

**CHIMP_1_235105 SOX-13 protein (Type 1 diabetes autoantigen ICA12) (Islet cell antigen 12) **				

184232055	-	HUMAN_1_850979	134.55	SOX-13 protein (Type 1 diabetes autoantigen ICA12) (Islet cell antigen 12)
-	-	CHIMP_1_235105	55.51	SOX-13 protein (Type 1 diabetes autoantigen ICA12) (Islet cell antigen 12)

**CHIMP_X_104156 ADP-ribosylation factoR-like protein 13A **				

100539703	-	HUMAN_X_788396	112.96	ADP-ribosylation factoR-like protein 13A
100539703	-	HUMAN_X_788399	106.14	ADP-ribosylation factoR-like protein 13A
-	-	CHIMP_X_104156	97.94	ADP-ribosylation factoR-like protein 13A
100539703	-	MONK_X_10115	93	ADP-ribosylation factoR-like protein 13A

**HUMAN_1_851772 Complement receptor type 1 precursor (c3b/C4b receptor) (CD35 antigen) **				

-	-	HUMAN_1_851772	90.18	Complement receptor type 1 precursor (c3b/C4b receptor) (CD35 antigen)
-	-	CHIMP_1_235873	86.46	Complement receptor type 1 precursor (c3b/C4b receptor) (CD35 antigen)
-	-	CHIMP_1_235895	86.46	Pan troglodytes mrna sequence, 3′ end of ORF
205746964	-	CHIMP_1_235891	85.81	Complement receptor type 1 precursor (c3b/C4b receptor) (CD35 antigen)
-	-	MONK_1_75296	76.91	Complement receptor type 1 precursor (c3b/C4b receptor) (CD35 antigen)
-	-	HUMAN_1_851821	75.98	Complement C4b binding protein CR-1 like (Fragment)
205746964	-	CHIMP_1_235905	74.1	Pan troglodytes mrna sequence, 3′ end of ORF
205746964	-	CHIMP_1_235909	74.1	Pan troglodytes mrna sequence, 3′ end of ORF

**HUMAN_1_851437 solute carrier family 26, member 9 isoform A **				

204150951	-	CHIMP_1_235519	351.23	Solute carrier family 26, member 9 isoform A
204150951	-	MONK_1_76747	337.22	Solute carrier family 26, member 9 isoform A
-	-	HUMAN_1_851439	252.06	Solute carrier family 26, member 9 isoform A

**HUMAN_1_837766 calcium activated chloride channel 3 precursor **				

-	86881166	CHIMP_1_224547	183.12	PREDICTED: Pan troglodytes similar to calcium—activated chloride channel
-	-	HUMAN_1_837766	157.82	Calcium activated chloride channel 3 precursor
-	86881166	COW_3_170345	139.02	Chloride channel, calcium activated, family member 3
-	86881166	COW_3_170045	138.65	Epithelial chloride channel protein (Calcium—activated chloride channel)
-	86881166	DOG_6_162589	128.97	∖N
-	86881166	MOUSE_3_2282090	123.94	Chloride channel calcium activated 4
-	86881166	RAT_2_220515	118.93	Calcium—activated chloride channel
-	86881166	OPOSSUM_2_28758	118.77	Calcium activated chloride channel 3 precursor
-	86881166	OPOSSUM_2_28690	116.67	Calcium activated chloride channel 3 precursor
-	86881166	RAT_2_220459	114.28	Chloride channel calcium activated 2
-	86881166	MOUSE_3_2281377	114.18	Chloride channel calcium activated 1
-	86881166	MOUSE_3_2281850	110.24	Chloride channel calcium activated 2
-	86881166	COW_3_170620	110.22	Hypothetical protein (Fragment)
-	86881166	RAT_2_220748	109.88	∖N
-	86881166	MONK_1_36555	103.72	Calcium activated chloride channel 4
-	86881166	CHIMP_1_224513	103.1	Chloride channel, calcium activated, family member 1 precursor
-	86881166	HUMAN_1_837721	103.1	Chloride channel, calcium activated, family member 1 precursor
-	86881166	MOUSE_3_2282601	102.32	Chloride channel calcium activated 3
-	86881166	MONK_1_36497	102.05	Calcium—activated chloride channel family member 1
-	86881166	COW_3_170508	100.32	Hypothetical protein LOC507504
